# Testing the psychometric properties of Kidscreen-27 with Irish children of low socio-economic status

**DOI:** 10.1007/s11136-016-1432-1

**Published:** 2016-10-18

**Authors:** Stephen Shannon, Gavin Breslin, Ben Fitzpatrick, Donncha Hanna, Deirdre Brennan

**Affiliations:** 1 Sport and Exercise Sciences Research Institute, Ulster University, Jordanstown, BT37 OQB Northern Ireland, UK; 2 School of Psychology, Queen’s University Belfast, Belfast, BT9 5BN Northern Ireland, UK

**Keywords:** Health-related quality of life, Children, Confirmatory factor analysis, Socio-economic status

## Abstract

**Background:**

Kidscreen-27 was developed as part of a cross-cultural European Union-funded project to standardise the measurement of children’s health-related quality of life. Yet, research has reported mixed evidence for the hypothesised 5-factor model, and no confirmatory factor analysis (CFA) has been conducted on the instrument with children of low socio-economic status (SES) across Ireland (Northern and Republic).

**Method:**

The data for this study were collected as part of a clustered randomised controlled trial. A total of 663 (347 male, 315 female) 8–9-year-old children (*M* = 8.74, SD = .50) of low SES took part. A 5- and modified 7-factor CFA models were specified using the maximum likelihood estimation. A nested Chi-square difference test was conducted to compare the fit of the models. Internal consistency and floor and ceiling effects were also examined.

**Results:**

CFA found that the hypothesised 5-factor model was an unacceptable fit. However, the modified 7-factor model was supported. A nested Chi-square difference test confirmed that the fit of the 7-factor model was significantly better than that of the 5-factor model. Internal consistency was unacceptable for just one scale. Ceiling effects were present in all but one of the factors.

**Conclusions:**

Future research should apply the 7-factor model with children of low socio-economic status. Such efforts would help monitor the health status of the population.

## Introduction

Health-related quality of life (HRQOL) is a multi-dimensional construct and refers to physical, psychological, social and behavioural components of children’s well-being [1]. As the United Kingdom (UK) Government include well-being as a marker of health [2], HRQOL measures can be used to assess the health status of the population. Hence, valid and reliable instruments are required [3]. Confirmatory factor analysis (CFA) is considered a robust analytic method to explain and establish construct validity among observed and unobserved (latent) variables [4] within such instruments. Kidscreen-27 [5] is a HRQOL measure that is applied in behavioural science research with children.

According to Schreiber et al. [6], when observed and latent variables are strongly correlated, the more likely it is that the instrument is reliable. As sample characteristics can be diverse and include various ages, cultures, genders and social groups, Park et al. [7] suggested that researchers should account for group differences in measurement selection in order to prevent potential confounders that may lead to flawed conclusions. Therefore, it is desirable to conduct CFA in cases where data from specific populations have not undergone instrument reliability and validity testing [8].

The Kidscreen HRQOL model was developed as part of the first cross-cultural, European-based attempt to standardise the measurement of children’s HRQOL [1]. In the instrument development phase, the Kidscreen group conducted focus groups with children, a Delphi procedure with experts, and item reduction and CFA techniques [9]. As traditionally children’s HRQOL measures have been developed through expert opinion [9], this method ensured that the content and language of the Kidscreen questionnaires was consistent with how children interpret HRQOL as a concept. Subsequently, CFA of a 52-item questionnaire was tested in a study of 22,827 children across 13 European countries and resulted in an initial 10-dimensional factor structure [10]. Further analysis [11] resulted in a reduced 27-item measure that is embedded within Kidscreen-52. The Kidscreen instruments are now widely used to monitor the health status of both healthy and unhealthy children aged 8–18 [1].

In Kidscreen-27, HRQOL is hypothesised to consist of a 5-dimensional structure: *physical well*-*being, psychological well*-*being, social support and peers, parent relations and autonomy, and school environment* (see Table 1). Yet, the 5-factor structure of Kidscreen-27 has received mixed support with some studies finding a good [11–13] and unacceptable fit with modifications for a 7-factor structure [14]. Although one of these studies [11] included children from the Republic of Ireland, the socio-economic status (SES) of these participants was not reported. Moreover, in Northern Ireland, Kidscreen-27 has only been tested online using exploratory factor analysis methods [15]. Researchers [4, 7, 8] propose that psychometric models should be tested in multiple populations to determine whether the hypothesised structure is generalisable. Hence, it is necessary to conduct CFA on Kidscreen-27 to determine whether: (a) researchers should opt for the hypothesised 5-factor [11] or modified [14] 7-factor structure; and (b) whether Kidscreen-27 is a psychometrically consistent measure of HRQOL for children of low SES across both Irish jurisdictions (Northern and Republic).

**Table 1:** Scale description detailing the conceptual themes for Kidscreen-27 5- and 7-factor models

	Description
Kidscreen-27 5-factor dimension	
Physical health (5 items)	This dimension explores the children’s perceptions of their physical activity, health and vitality. It includes items that refer to the children’s energy and their ability to physically function
Psychological well-being (7 items)	This dimension explores the children’s experiences of positive and negative affect. Items are worded to reflect the children’s mood, enjoyment and experiences of happiness and loneliness
Parent relations and autonomy (7 items)	This dimension considers the children’s relationships with their parents, availability of free time and satisfaction with their financial resources
Social support and peers (4 items)	This dimension examines the quality of the children’s interactions with their peers. Levels of trust as well as time spent with peers are considered
School environment (4 items)	This dimension reflects children’s perceptions of their attention, experience of school and relationship with teachers
Kidscreen-27 7 factor dimension	
Physical health (5 items)	This dimension explores the children’s perceptions of their physical activity, health and vitality. It includes items that refer to the children’s energy and their ability to physically function
Psychological well-being (4 items)	This dimension explores the children’s experiences of enjoyment and positive affect
Moods and emotions (3 items)	This dimension reflects the children’s experiences of depressive moods and emotions
Parent relations and autonomy (5 items)	This dimension considers the children’s relationships with their parents and availability of free time
Financial resources (2 items)	This dimension asks the children whether they are satisfied with their financial resources
Social support and peers (4 items)	This dimension examines the quality of the children’s interactions with their peers. Levels of trust as well as time spent with peers are considered
School environment (4 items)	This dimension reflects children’s perceptions of their attention, experience of school and relationship with teachers

The objective of this study is to present the psychometric properties of Kidscreen-27 with Irish children of low SES. The purpose of testing the instrument is to determine whether the measure is conceptually valid and internally consistent for assessing HRQOL with children. This evidence presented below recommends how future research can monitor the health status of the population using Kidscreen-27.

## Methods

### Participants

The data for this study were collected as part of a clustered randomised control intervention study, Sport for LIFE: All Island (SFL:AI). A sample of 705 children (364 boys) aged 8–9 years (*M* = 8.74, SD = .50) took part during September 2014 and January 2015, corresponding to the delivery of the intervention across Ireland. Due to missing data analysis (outlined below), 94 % (663 children, 347 male, 315 female) of the total SFL:AI sample was included in the final analyses. Geographically, the sample consisted of 186 children from Northern Ireland and 477 from the Republic of Ireland.

### Recruitment and procedure

Ethical approval was obtained from Ulster University’s Research Ethics Committee. In both jurisdictions (Northern Ireland and Republic of Ireland), participants were recruited from urban schools in areas of social and economic disadvantage based on deprivation indexes. The Multiple Deprivation Measure in Northern Ireland consists of seven domains of deprivation: income, employment, health, education, proximity to services, living environment and crime. This database gives a living area, as indicated by postcode, a Super Output Area (SOA) rank, with one being the lowest. Schools situated in the most deprived areas were identified. In the Republic of Ireland, only schools identified by Department of Education’s [16] Delivering Equality of Opportunity in Schools (DEIS) programme were included. The DEIS database includes socio-economic variables such as local authority accommodation, lone parenthood, travellers, large families (five or more children) and pupils eligible for free books. Studies [17, 18] show that pupils attending schools listed on the NISRA and DEIS databases are likely to be from lower social class backgrounds and have parents with fewer educational qualifications. A sample of the schools (*n* = 27) was chosen via a manual random number generator and invited to participate in SFL:AI.

School principals were contacted, and all agreed to participate and distributed information sheets about the study to the classroom teachers and to children’s parents. Only participants who provided written assent and consent from their parents participated in the study. To ensure anonymity, participants were given a unique code for the questionnaire. The questionnaires were administered to the participants under quiet classroom conditions. Instructions and information regarding the completion of the questionnaire were explained by a lead researcher, and minor details such as word pronunciation were described to the children in groups of 5–10 with a trained researcher accompanying each group.

### Outcome measures

#### Health-related quality of life

HRQOL was measured using the 27-item questionnaire, Kidscreen-27 [5]. Kidscreen-27 has five dimensions (see Table 1 for description and Table 4 for wording of individual items). The measure was developed through exploratory factor analysis, regression and item reduction techniques, and confirmatory factor analysis [1]. Items are rated on a 5-point Likert scale ranging from 1 = never, 2 = seldom, 3 = quite often, 4 = very often and 5 = always—reflecting the frequency of behaviours or feelings; or 1 = not at all, 2 = slightly, 3 = moderately, 4 = very, 5 = extremely—reflecting the intensity of a belief or attitude in the previous week.

### Data management

Raw scores from the questionnaire were entered into Statistical Package for Social Sciences [19]. Three items (no. 9, 10 and 11) required reverse scoring for negatively worded responses. To screen for missing data, Little’s Missing Completely at Random (MCAR) [20] test was run on each subscale. According to Little’s [20] MCAR test, if the Chi-square statistic is not statistically significant, the data are missing in random order. The data were missing at random for each scale (*p* > .05). As such, 6 % of the sample had complete missing data on a given scale. Chi-square tests confirmed that there were no significant demographic differences between those participants with missing data and those with non-missing data. To this end, the first author analysed the patterns of missing data and conducted a listwise deletion of these participations (*n* = 42).

The expectation–maximisation (EM) algorithm was used for the maximum likelihood (ML) estimation of the remaining missing data. The EM method estimates a score for missing data based on three steps: first, the means, variances and covariances for complete data sets are estimated; second, regression formulas are created based on these estimations; and third, these formulas are used to estimate missing scores [21]. EM was conducted on each individual scale to use data, in a theoretical sense, from inter-correlated items as predictors of the missing data [22]. On average, 3.5 % of the data were estimated for each scale.

### Data analysis

Descriptive statistics were calculated. The items from each scale were added together to represent a total mean score and standard deviation for each factor. Further, in accordance with the Kidscreen scoring methodology [1], we also transformed the 5-factor scale scores into standardised *T* values and standard deviations so that other studies [11–14] can directly compare their results with the present study. Ceiling and floor effects were also calculated and determined present if more than 15 % of the sample scored the highest or lowest score, respectively [23]. Cronbach’s alpha was calculated as a measure of internal consistency. However, as there are fewer than 5 items on each scale, we approached this value with caution and considered an alpha coefficient value of .60 or above as acceptable [24].

For the demographic variable gender (females or males), we conducted a series of one-way between-group analyses of variance (ANOVAs) to test for significant differences between the groups on each of the original and modified factors. Alpha significance was set to *p* < .05, and partial eta squared (*η*_*p*_^2^) was calculated as a measure of effect size.

Mardia’s [25, 26] coefficient of multivariate kurtosis and its critical ratio of 1.96 were performed to test the data for multivariate normality. The principle of multivariate kurtosis is to either: (a) ensure normality before conducting conventional statistical hypothesis testing or (b) reject normality and conduct tests with bootstrapping procedures for non-normal data [27, 28]. As the data departed from normality in all of the models, the alpha significance value was corrected for bias at the 95 % confidence interval, and Bollen–Stine bootstrapping was conducted with 1000 samples in order to have improved accuracy when assessing parameter estimates and fit indices [27].

The factor structure of Kidscreen-27 was tested through CFA. Models were specified using AMOS [29] using the maximum likelihood estimation. A CFA was conducted on the original hypothesised 5-factor model [1], and modified 7-factor model [14] of the Kidscreen-27 measure, based upon mixed success for the original 5-factor model [14].

**Fig. 1:**
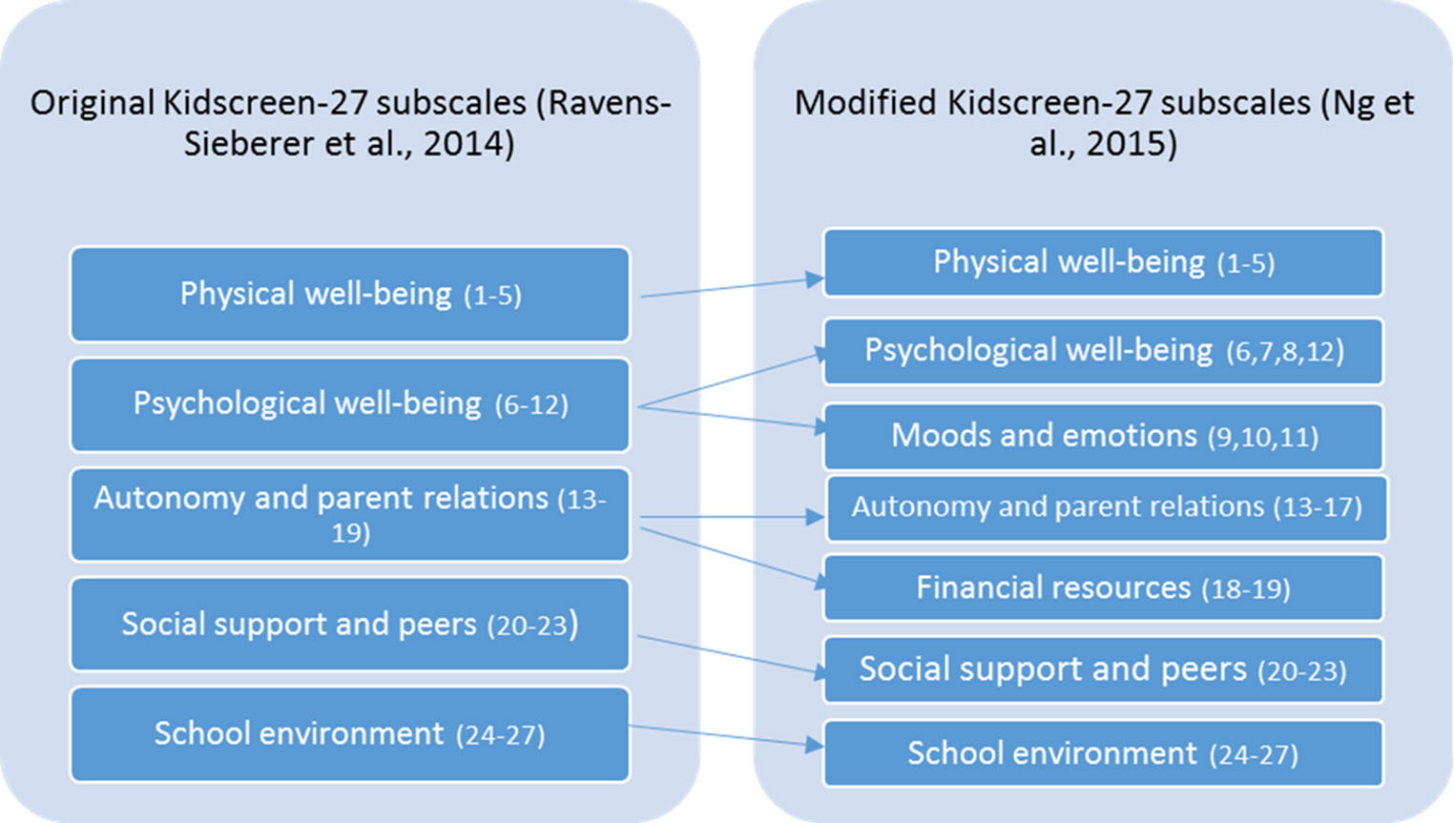
Dimensionality map for 5- and 7-factor Kidscreen-27 models. *Note asterisk* (*) item numbers in brackets

Specifically, this involved splitting the psychological well-being and autonomy, and parent relations factors into two further factors (see Fig. 1 for dimensionality map and Table 1 for information on the adjusted scale description). The psychological well-being factor was split as focus group research [9] shows that children’s psychological well-being encompasses both positive and negative dimensions. Four items (6, 7, 8 and 12) represent positive perceptions and emotions, while three items (9, 10 and 11) represent depressive moods and emotions. The autonomy and parent relations factor was split as previous research [14] found that the two items specifically related to financial resources were tapping into an independent financial resources factor, as specified in the 52-item model [1]. Therefore, this produced an autonomy and parent relations factor [5 items] and financial resources factor [2 items]. Consistent with the structure of the Kidscreen instruments, all the factors were allowed to correlate [11], and a correlation matrix was calculated for the 5- and 7-factor models, considering positive values of .0 to .3 as weak, .31 to .70 as moderate and .71 or above as strong [22].

The adequacy of the models was assessed using multiple goodness-of-fit indices. The Chi-square (*χ* ^2^) goodness-of-fit index was reported with a small non-significant *χ* ^2^ statistic indicating good model fit. We approached this value with caution given that large sample sizes tend to result in statistically significant Chi-square values [8]. The comparative fit index (CFI), the Tucker-Lewis Index (TLI) and the goodness-of-fit index (GFI) were reported with values of >.90 [30] or >.95 [27], considered as acceptable or good model fit, respectively. The root-mean-square error of approximation (RMSEA) was reported as a badness of fit index, with values of close to .06 or below considered acceptable [31]. As the models are nested, a Chi-square test of independence was conducted to compare the fit of the 5- and 7-factor models.

## Results

Descriptive statistics are presented in Table 2. Cronbach’s alpha values ranged from .56 to .74. The moods and emotions scale in the 7-factor model was the only subscale below the recommended value of .6. No floor effects were observed, but ceiling effects were observed on all of the factors aside from the autonomy and parental relations scale in 5-factor version of Kidscreen-27.

**Table 2:** Descriptive statistics for total raw and *T* scale means, standard deviation, internal consistency and floor and ceiling effect

Scale	Items	Raw *M*	Raw SD	*T* value *M*	*T* value SD	*α*	Floor (%)	Ceiling (%)
Kidscreen-27 5-factor								
Physical well-being	5	19.48	2.93	56.37	10.38	.65	.2	15.5
Psychological well-being	7	31.26	3.74	52.19	10.52	.65	0	16.4
Autonomy and parent relations	7	28.67	4.82	52.19	10.52	.66	0	9.7
Social support and peers	4	17.87	2.90	56.42	10.56	.74	.6	40.3
School environment	4	17.41	3.02	58.97	10.88	.72	.2	30.8
Kidscreen-27 7-factor								
Physical well-being	5	19.48	2.93	n/a	n/a	.65	.2	15.5
Psychological well-being	4	19.00	2.40	n/a	n/a	.63	.2	31.4
Moods and emotions	3	13.19	2.23	n/a	n/a	.56	.3	36.8
Autonomy and parent relations	5	21.24	3.40	n/a	n/a	.60	.2	18.6
Financial resources	2	7.42	4.23	n/a	n/a	.69	5.8	27.1
Social support and peers	4	17.87	2.90	n/a	n/a	.74	.6	40.3
School environment	4	17.41	3.02	n/a	n/a	.72	.2	30.8

Raw *M*, raw mean score; Raw SD, standard deviation for raw mean score; *T* value *M*, *T* value mean; *T* value SD, standard deviation for *T* score; α, Cronbach’s alpha value; and n/a, non applicable

A series of one-way between-groups ANOVA statistical tests revealed no significant differences between gender on the physical well-being, original and modified psychological well-being, moods and emotions, and social support and peers factors. However, females (*M* 29.12; SD 4.65) scored significantly higher than males (*M* 28.26; SD 4.93) on the original parent relations and autonomy dimension in the 5-factor model [*F* (2, 661) = 5.33, *p* < .05; *η*_*p*_^2^ = .008]. When we analysed this factor as two distinct dimensions (as specified in the 7-factor model), females (*M* 21.61; SD 3.19) scored significantly higher than males (*M* 20.91; SD 3.54) on the modified parent relations and autonomy dimension [*F* (2, 661) = 7.00, *p* < .01; *η*_*p*_^2^ = .01], but not on the financial resources dimension. The significance level and effect size were larger for the modified parent relations and autonomy dimension, in comparison with the original. Females (*M* 18.02; SD 2.52) also scored significantly higher than males (*M* 16.86; SD 3.32) on the school environment factor [*F* (2, 661) = 24.78, *p* < .001; *η*_*p*_^2^ = .03].

The fit indices and factor loadings for the two models are presented in Tables 3 and 4. All factor loadings were statistically significant (*p* < .05). The fit indices showed that the original 5-factor Kidscreen-27 model was an unacceptable fit, with the CFI and TLI values being below .9, and the *χ* ^2^ value was statistically significant (*p* < .01). However, the RMSEA was acceptable. This model was subsequently rejected in favour of the modified 7-factor Kidscreen-27 model as this yielded acceptable-to-good fit indices for CFI, TLI and RMSEA. A nested Chi-square difference test comparing the two models confirmed that the fit of the 7-factor model was significantly better than that of the 5-factor model [*χ* ^2^ (11) = 258.916, *p* < .01]. The correlation matrix for the 5- and 7-factor models is presented in Tables 5 and 6. For both models, the correlations between the factors were all positive and significant (*p* < .05). The factors in the 5-factor model were all moderately correlated, while the correlations between factors in 7-factor model ranged from weak, moderate to strong.

**Table 3:** Summary of fit indices for Kidscreen 5- and 7-factor models

Model	*df*	*χ* ^2^ (*p*)	CFI	GFI	TLI	RMSEA
Kidscreen-27						
5-Factor model	314	793.005, (*p* < .01)	.863	.917	.847	.048 (90 % CI .044–.052)
7-Factor model	303	534.089, (*p* < .01)	.934	.944	.924	.034 (90 % CI .029–.039)

**Table 4:** Individual item factor loadings for Kidscreen 5- and 7-factor models

Item number and description	5-Factor model		7-Factor model	
	Subscale	Factor loading	Subscale	Factor loading
1: In general, how would you say your health is?	PH	.50	PH	.50
2: Have you felt fit and well?	PH	.61	PH	.60
3: Have you been physically active?	PH	.49	PH	.49
4: Have you been able to run well?	PH	.48	PH	.49
5: Have you felt full of energy?	PH	.60	PH	.60
6: Has your life been enjoyable?	PsyWB	.60	PsyWB	.60
7: Have you been in a good mood?	PsyWB	.63	PsyWB	.63
8: Have you had fun?	PsyWB	.52	PsyWB	.52
9: Have you felt sad?	PsyWB	.36	M & E	.46
10: Have you felt so bad that you didn’t want to do anything?	PsyWB	.32	M & E	.56
11: Have you felt lonely?	PsyWB	.26	M & E	.48
12: Have you been happy with way you are?	PsyWB	.46	PsyWB	.61
13: Have you had enough time for yourself?	Parent and autonomy	.40	Parent and autonomy	.44
14: Have you been able to do the things that you want to do in your free time?	Parent and autonomy	.46	Parent and autonomy	.47
15: Have your parent(s) had enough time for you?	Parent and autonomy	.49	Parent and autonomy	.52
16: Have your parent(s) treated you fairly?	Parent and autonomy	.41	Parent and autonomy	.44
17: Have you been able to talk to your parent(s) when you wanted to?	Parent and autonomy	.49	Parent and autonomy	.53
18: Have you had enough money to do the same things as your friends?	Parent and autonomy	.54	Financial resources	.78
19: Have you had enough money for your expenses?	Parent and autonomy	.54	Financial resources	.67
20: Have you spent time with your friends?	Social support	.66	Social support	.66
21: Have you had fun with your friends?	Social support	.74	Social support	.74
22: Have you and your friends helped each other?	Social support	.68	Social support	.68
23: Have you been able to rely on your friends?	Social support	.56	Social support	.56
24: Have you been happy at school?	School environment	.61	School environment	.61
25: Have you got on well at school?	School environment	.67	School environment	.67
26: Have you been able to pay attention?	School environment	.62	School environment	.62
27: Have you got along well with your teachers?	School environment	.65	School environment	.65

*PH* physical health, *PsyWB* psychological well-being, *M* & *E* moods and emotions

**Table 5:** Correlation matrix for the Kidscreen 5-factor model

Factor	Correlation				
Physical well-being	1				
Psychological well-being	.68	1			
Parent relations and autonomy	.47	.61	1		
Social support and peers	.32	.56	.55	1	
School environment	.39	.64	.43	.35	1

**Table 6:** Correlation matrix for the Kidscreen 7-factor model

Factor	Correlation						
Physical well-being	1						
Psychological well-being	.74	1					
Moods and emotions	.21	.50	1				
Parent relations and autonomy	.43	.65	.45	1			
Financial resources	.37	.29	.27	.51	1		
Social support and peers	.32	.57	.30	.49	.44	1	
School environment	.39	.64	.35	.46	.22	.35	1

## Discussion

Kidscreen-27 is a child-centred, cross-cultural instrument that can monitor the HRQOL of children and provide clarity on which aspects of children’s environments may be salient for promoting health and well-being. The purpose of this study was to determine the factor structure and internal consistency of Kidscreen-27 [1] with children of low SES across the island of Ireland. By including children of low SES from both Irish jurisdictions (Northern and Republic), this study extends previous work [11, 12, 14, 17] testing the factor structure of Kidscreen-27. CFA conducted on the two specified Kidscreen-27 models found that the modified 7-factor model [14] was a better explanation of the data than the original 5-factor [5] model. This was confirmed with a nested Chi-square difference test. Overall, these findings provide methodological recommendations for future HRQOL research with children.

The fit indices for the 5-factor model revealed unacceptable *χ* ^2^, CFI and TLI values. This finding contradicts a CFA conducted on Kidscreen-27 in 13 European countries [11] and with Chilean adolescents [32] that found an acceptable model fit. However, the sample characteristics were different. For example, Robital et al.’s [11] study used an aggregated score from both children’s and adolescents’ (aged 8–18) data, while the present study consisted exclusively of preadolescent children (aged 8–9). Confirmatory evidence for the modified 7-factor Kidscreen-27 model in this study supports recent research [14] and may offer methodological reasons for the unsuitability of the 5-factor model for younger children.

Specifically, this model split the psychological well-being subscale into two independent scales. Previous focus group research [9] has found that children’s psychological well-being has positive and negative dimensions. Although these dimensions are moderately correlated (as evidenced in the correlation matrix, see Table 6), meaning that children with positive emotions are less likely to experience negative emotions, findings from the CFA in this study suggest that two independent factors are required for conceptualising the psychological well-being construct in children. However, it is worth noting that the Cronbach’s alpha value was below the recommended limit for the modified moods and emotions scale that is constructed with negatively worded items. Previous research [33, 34] has argued against the use of negatively worded items as they are likely to reduce scale reliability and consistency. Indeed, research [35] with pre-adolescent children (aged 6–12) has found that negatively worded items can be problematic as younger children are just beginning to develop their reading skills and thus may find it difficult to interpret negatively worded items. Considering the mean age of this sample was 8.5 years, this may explain why the negatively worded items were found to have low internal consistency.

Akin with previous research [14], the autonomy and parent relations factor loaded onto two independent factors, with one relating to the children’s sense of autonomy and relationships with parents, and one relating to satisfaction with financial resources. However, the largest CFA of Kidscreen-27 [11] did not report this issue, despite the inter-factor correlations for the financial resources items being the lowest across the whole model. Statistical testing found that gender had no significant relationship with the modified financial resources factor. However, gender had a significant relationship with the original and modified parental relations and autonomy factor, with females scoring higher than males. Notably though, a stronger effect size and significance value was found between gender and the modified parental relations and autonomy factor, in comparison with the original. Moreover, the correlation matrix found just a moderate (.51) relationship between children’s parental relations and autonomy with their satisfaction with financial resources. Therefore, as children’s autonomy is only starting to form during pre-adolescence [36], their satisfaction with financial resources is linked with their parental relations, but our study suggests that they are independent of each other.

Ceiling effects were present in all but one of the subscales (autonomy and parent relations in 5-factor Kidscreen-27). Ceiling effects have been previously reported in the social support and peers and school environment scales with 10-year-old children [13], but not with 12-year-old children [9]. As the present study was the first to psychometrically test for floor and ceiling effects in Kidscreen-27 with younger children (aged 8–9), it is unclear whether younger children score higher on these indices of HRQOL. Future use of Kidscreen-27 with younger children should test for floor and ceiling effects and, if necessary, apply bootstrapping and nonparametric models that have been shown to correct for bias [22, 37].

## Conclusion

The strength of this study was a rigorous psychometric testing of Kidscreen-27 [5] with a large sample of Irish children from areas of low SES. Overall, the 7-factor model of Kidscreen-27 is suitable for assessing the HRQOL of pre-adolescent children (aged 8–10) of low SES. Future research should consider the linkages, but independence, between positive and negative psychological well-being dimensions; and between children’s parental relations and autonomy, and their satisfaction with financial resources. However, as the Cronbach’s alpha value was low for the moods and emotions factor, future research may consider constructing positively worded items to determine whether the internal consistency can be improved. Limitations of this study are that the test/retest reliability and convergent/divergent validity of Kidscreen-27 remain unassessed in this population. Longitudinal study designs examining HRQOL with multiple instruments may provide a more comprehensive psychometric assessment of Kidscreen-27 than the cross-sectional design conducted in the current study.
